# Agreement and Reliability of a Digital Incentive Spirometer Compared with a Volume-Oriented Incentive Spirometry Device Across Different Age Groups

**DOI:** 10.3390/bios16070361

**Published:** 2026-06-29

**Authors:** Kornanong Yuenyongchaiwat, Lucksanaporn Mahawong, Chaopraya Nenmanee, Sasipa Buranapuntalug, Chusak Thanawattano

**Affiliations:** 1 Physiotherapy Department, Faculty of Allied Health Sciences, Thammasat University, Pathum Thani 12120, Thailand; 2 Thammasat University Research Unit for Physical Therapy in Respiratory and Cardiovascular Systems, Thammasat University, Pathum Thani 12120, Thailand; 3 Biomedical Electronics and Systems Research Team, Assistive Technology and Medical Devices Research Group, National Electronics and Computer Technology Center (NECTEC), Pathum Thani 12120, Thailand

**Keywords:** digital health, healthcare innovation, incentive spirometer, medical device, inspiratory volume

## Abstract

Incentive spirometry is widely used in respiratory rehabilitation to enhance lung expansion and prevent postoperative pulmonary complications. However, conventional devices, including volume-oriented and flow-oriented incentive spirometers, rely on subjective visual interpretation, which may limit measurement accuracy and clinical utility. A digital incentive spirometer (DIS) has been developed to provide objective, real-time measurements of inspiratory volume. This study aimed to evaluate the agreement and reliability between the DIS and a volume-oriented incentive spirometer (VIS) across different age groups. A cross-sectional study was conducted in 150 participants aged 7–80 years, stratified into five age groups with equal sex distribution. Inspiratory volume was measured simultaneously using both devices. Agreement was assessed using Bland–Altman analysis, and reliability was evaluated using intraclass correlation coefficients (ICC). The DIS demonstrated good overall reliability (ICC = 0.868, 95% CI: 0.821–0.903). The mean difference was 48.69 mL, indicating slight overestimation by the DIS. However, the limits of agreement were wide (−469.24 to 566.63 mL), suggesting limited interchangeability. Reliability varied across age groups, with the highest ICC in older adults and the lowest in adolescents. The DIS showed good reliability but limited agreement with the VIS.

## 1. Introduction

Breathing exercises aimed at improving lung expansion and increasing lung volume can generally be classified into manual and device-assisted techniques. Manual breathing techniques primarily include diaphragmatic breathing, which emphasizes engaging the diaphragm to promote efficient ventilation, reduce the work of breathing, and enhance thoracoabdominal movement. Previous studies have demonstrated that diaphragmatic breathing improves respiratory muscle function, breathing efficiency, and pulmonary function in both healthy individuals and patients, e.g., respiratory disorders or cardiovascular disease [1,2,3,4,5].

In contrast, device-assisted breathing exercises commonly involve the use of an incentive spirometer, which is designed to encourage sustained maximal inspiration and facilitate lung expansion. Incentive spirometry has been widely used in clinical practice, particularly in postoperative care and pulmonary rehabilitation, to prevent or reduce pulmonary complications such as atelectasis and to promote deep breathing [6,7,8,9]. One of the major advantages of incentive spirometry is its ability to provide real-time visual feedback during the breathing maneuver. This visual feedback enables users to monitor their inspiratory performance, enhances motivation and adherence to the exercise program, and enhances the effectiveness of respiratory training by facilitating active patient participation. Consequently, incentive spirometry remains a popular and accessible tool for respiratory rehabilitation and lung expansion therapy.

Incentive spirometry is commonly used in pulmonary rehabilitation to promote lung expansion, prevent postoperative pulmonary complications, improve pulmonary function in patients with pulmonary disease, and improve gas exchange. The primary mechanism involves encouraging patients to perform slow, deep inhalations, which increase lung volume, enhance secretion clearance, and improve gas-exchange efficiency [10,11,12,13,14].

Conventional incentive spirometry devices are generally classified into two main types: volume-oriented and flow-oriented spirometers [15,16,17]. A volume-oriented incentive spirometer (VIS) is designed to promote lung expansion by increasing inspiratory volume, typically providing visual feedback through a piston that reflects the inhaled volume. In contrast, flow-oriented incentive spirometers (FIS) focus on controlling inspiratory flow and encourage participants to achieve target inspiratory effort levels [15,16,17]. These devices also provide feedback that allows patients to monitor their respiratory performance and progressively improve breathing patterns, thereby enhancing motivation and adherence to therapy [9,15,18]. In addition, flow sensors (i.e., FIS) and volume sensors (i.e., VIS) in incentive spirometers measure different aspects of inhalation to encourage deep breathing and lung expansion. Flow sensors focus on inhalation speed based on inspiratory airflow, while volume sensors track total air inhaled [17].

Despite their widespread clinical use, these devices commonly rely on subjective visual interpretation and have limited display resolution, which may reduce measurement precision and limit objective monitoring during pulmonary rehabilitation. The conventional FIS relies on visual observation of three chambers in a row containing a ball, each corresponding to inspiratory flow levels equivalent to approximately 600, 900, and 1200 mL per second, respectively [16]. However, this design presents several limitations. Due to the discrete nature of the measurement, it may not accurately capture inspiratory performance in patients with severe respiratory impairment who are unable to generate sufficient inspiratory effort to elevate any of the balls. Furthermore, the reliance on subjective visual assessment limits the precision and reproducibility of measurements. Healthcare providers may be unable to consistently record exact values or monitor subtle changes over time. This lack of quantitative accuracy can affect clinical decision-making, as well as the evaluation of patient progress during pulmonary rehabilitation. This limitation may lead to an underestimation of inspiratory capacity in patients with severe respiratory dysfunction and reduce the sensitivity of the device in detecting clinically meaningful changes over time.

To address these limitations, a digital incentive spirometer (DIS) was developed in the present study. The DIS was designed using a differential pressure-based flow sensing system integrated with microcontroller-based signal processing. The DIS operates on a sensor system that detects airflow and timing during inspiration. The airflow signal is continuously recorded and plotted as a function of time, allowing for the calculation of inspiratory volume by determining the area under the flow–time curve [19]. The analog signals obtained from the sensor are processed through an analog-to-digital converter and subsequently analyzed by a microcontroller. The computed inspiratory volume is then displayed in a digital format on the device interface. Further, the DIS has the potential to improve both the sensitivity of respiratory assessment and the overall quality of patient management in clinical practice [20].

We thereby developed the DIS, named Breathive, which enables users to accurately quantify inspiratory volume, even at low levels of respiratory effort. This feature is particularly beneficial for participants with limited inspiratory capacity, as it allows detection of small but clinically meaningful changes in lung function. In addition, the display presents numerical values corresponding to the achieved inspiratory volume, enabling precise and objective measurement [21]. In addition, the Breathive has been used on patients with open heart surgery compared to FIS, and it has been reported that both devices improve pulmonary function and respiratory muscle strength [21]. However, the Breathive has not yet been compared with a clinically used VIS across multiple age groups ranging from children to older adults. This approach provides important information regarding the clinical applicability, measurement characteristics, and age-related performance. Therefore, this study aimed to evaluate the agreement and reliability of the developed DIS, named Breathive, in comparison with a commercially available VIS, the Voldyne incentive spirometer, which provides quantitative measurements of inspiratory volume. It was hypothesized that the developed DIS would demonstrate good agreement and high reliability when compared with the commercially available VIS for measuring inspiratory volume. The findings of this study are expected to provide evidence supporting the use of Breathive as a valid and reliable DIS for inspiratory volume measurement. In addition, Breathive may offer practical advantages in pulmonary rehabilitation by providing real-time visual feedback, which could enhance patient motivation, adherence to breathing exercises, and clinical monitoring of respiratory performance in patients of all ages.

## 2. Materials and Methods

### 2.1. Participants

A cross-sectional comparative design was used to evaluate agreement and reliability between a Breathive and the Voldyne^®^ 4000 (Hudson RCI Voldyne, Teleflex Medical, Morrisville, NC, USA) incentive spirometer, a volume-oriented device that displays the inspiratory volume.

A sample size of 150 participants was considered adequate based on previous methodological recommendations, which suggest that at least 50–100 subjects are required to obtain stable estimates of reliability and agreement [22,23,24]. Age was treated as a categorical variable because the primary aim of the study was to evaluate device agreement and reliability within predefined clinically relevant age groups. Participants aged 7 to 80 years were recruited and stratified into five age groups: childhood (7–12 years), adolescence (13–17 years), young adulthood (18–35 years), middle adulthood (36–59 years), and older adulthood (60–80 years). These groups were selected to represent clinically relevant developmental and physiological stages across the lifespan. In addition, each group had an equal sex distribution, comprising 15 males and 15 females, and ensured balanced representation throughout the lifespan. All participants were assessed under the same experimental conditions. A normal body mass index (18.5–24.9 kg/m^2^), a resting heart rate lower than 120 beats per minute, and controllable blood pressure were included. Participants who had a current respiratory infection, current acute chest pain, and history of mental health problems were excluded.

### 2.2. Instrument

The Breathive was developed based on the principle of differential pressure flow measurement. A pressure sensor (MPX10DP, Freescale Semiconductor, Austin, TX, USA) was used to detect pressure differences and generate an analog signal, which was amplified using an operational amplifier (LM358N, Texas Instruments, Dallas, TX, USA) and processed by an ESP32 microcontroller (Espressif Systems, Shanghai, China). Airflow measurements were calibrated against a standard airflow sensor (AWM720P1, Honeywell International Inc., Charlotte, NC, USA), known for its high accuracy and low measurement error. A linear relationship between pressure and airflow was established by simultaneously recording data from both sensors. The system converts pressure signals into airflow values and calculates inspiratory volume through analog-to-digital conversion and microcontroller processing. Airflow through the device, influenced by variations in cross-sectional area and pressure, is ultimately used to determine the volume of air inhaled into the lungs. The detail has been published elsewhere [21].

**Figure 1:**
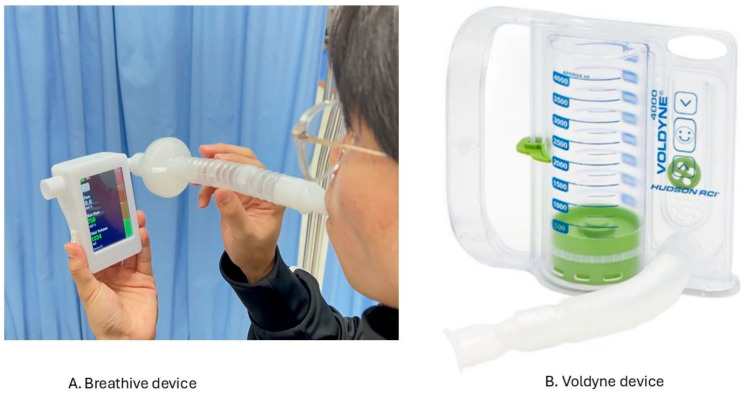
Comparison of Breathive and volume-oriented incentive spirometer.

The Breathive provides objective, real-time measurements of inspiratory volume (mL), flow rate, and peak inspiratory flow (mL/s), along with visual and auditory feedback to enhance patient performance. Additionally, the inclusion of a high-efficiency bacterial filter (99.99%) supports infection control and facilitates multi-patient use in clinical settings (Figure 1A).

The Voldyne^®^ 4000 incentive spirometer is the VIF, which measures inspired air volume. The feature display reading typically ranges between 250 and 4000 mL (Figure 1B). The Voldyne incentive spirometer was used according to the manufacturer’s specifications throughout the study.

### 2.3. Procedure

**Figure 2:**
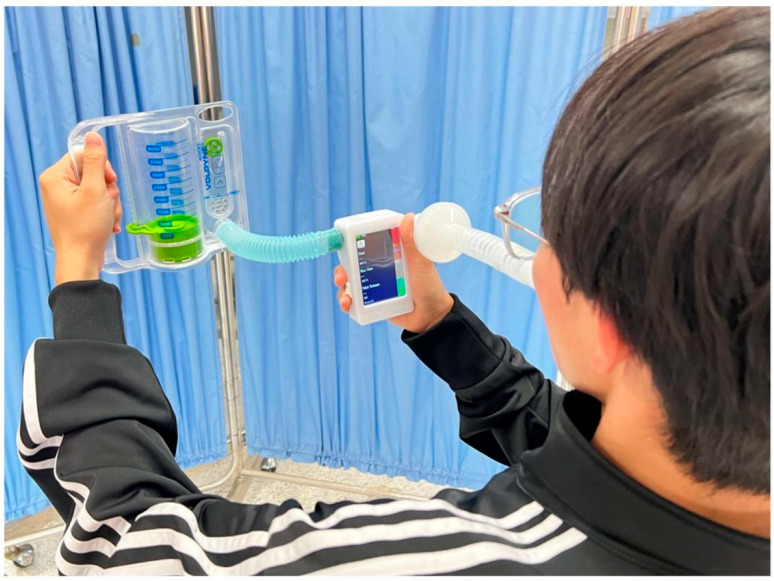
Validation setup of the Breathive device and the Voldyle device.

Participants were requested to sit in an upright position with the back supported. The Breathive was connected to a bacterial filter to minimize contamination and then attached to a mouthpiece. The opposite end of the Breathive was connected to the Voldyne device, allowing simultaneous measurement of inspiratory volume from both devices during each maneuver (Figure 2). During testing, the participant inhaled through a mouthpiece connected to the Breathive device, while the opposite side of the Breathive device was connected directly to the Voldyne device in a serial configuration. This arrangement allowed simultaneous inspiratory measurements from both devices during the same inspiratory maneuver. Participants were instructed to perform a slow, deep inhalation to maximal capacity through the mouthpiece. During each maneuver, inspiratory volume was simultaneously recorded by both the Breathive and the Voldyne. Each participant performed two trials, with a rest interval of approximately 1–3 s between attempts. The highest value obtained from the two trials was selected for analysis. To reduce observer bias, two independent assessors recorded the measurements. One assessor recorded the inspiratory volume displayed on the Breathive, while the other recorded the value from the Voldyne device. Both assessors were blinded to each other’s measurements.

### 2.4. Statistical Analysis

Agreement between the two devices was assessed using Bland–Altman analysis, including calculation of the mean difference (bias) and 95% limits of agreement (LoA). Reliability was evaluated using the intraclass correlation coefficient (ICC) based on a two-way random-effects model with absolute agreement. Subgroup analyses were conducted by age group to assess whether agreement and reliability varied across age categories. ICC less than 0.50 can be interpreted as poor, 0.50–0.75 is defined as moderate, 0.75–0.90 is identified as good, and >0.90 indicates excellent agreement [25]. Statistical significance was set at *p* < 0.05.

## 3. Results

Participants were enrolled in five age groups: 7–12 years, 13–17 years, 18–35 years, 36–59 years, and 60–80 years, with an equal number of male and female participants in each group. Inspiratory lung volume increased progressively from the 7–12 years age group to adolescence (13–17 years), reaching its peak in early adulthood (18–35 years). Therefore, lung volume gradually declined in the older age groups (36–59 and 60–80 years). This trend was consistently observed across measurements obtained from both the Breathive and the Voldyne device (Table 1).

A total of 150 participants were included in the analysis. Overall, the Breathive device demonstrated good reliability compared with the standard Voldyne device, with an ICC of 0.868 (95% CI: 0.821–0.903). Bland–Altman analysis showed a mean difference of 48.69 mL, indicating a slight overestimation by the Breathive device. However, the limits of agreement were wide (−469.24 to 566.63 mL), suggesting considerable variability between the two measurement methods.

**Figure 3:**
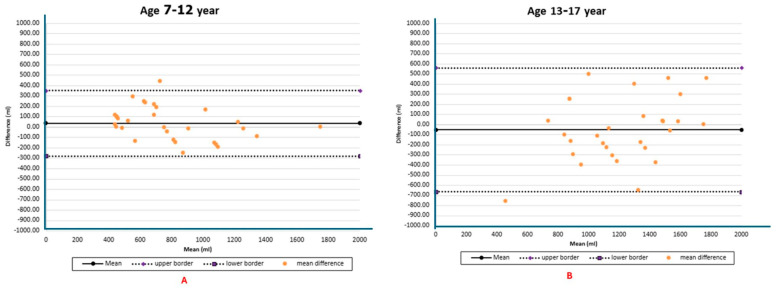

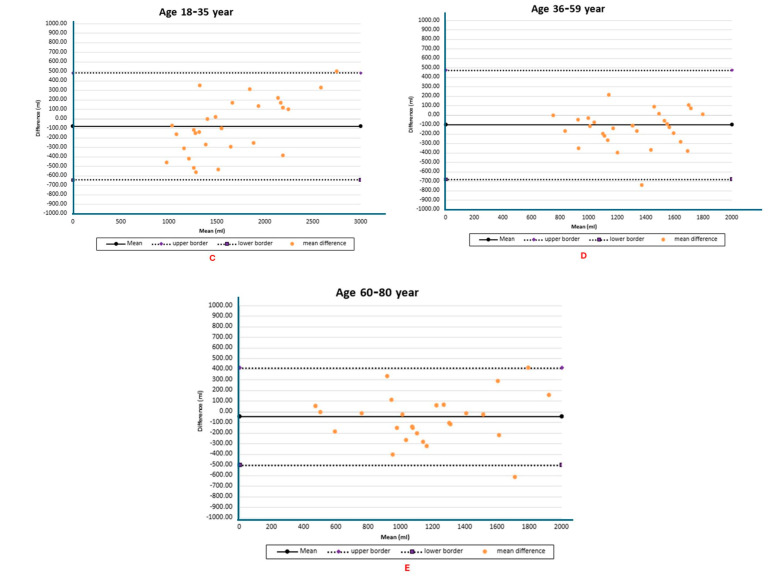
Bland–Altman plot of the inspiratory volume across different age groups. (**A**) age 7–12 years; (**B**) age 13–17 years; (**C**) age 18–35 years; (**D**) age 36–59 years; (**E**) age 60–80 years.

Subgroup analysis by age revealed variability in reliability and agreement. The highest reliability was observed in the 60–80 years group (ICC = 0.909), followed by the 7–12 years group (ICC = 0.882). In contrast, the 13–17 years group demonstrated the lowest reliability (ICC = 0.619). The mean difference was smallest in the 7–12 years group (−36.53 mL), indicating slight underestimation, whereas larger positive biases were observed in older age groups, particularly in the 36–59 years group (102.60 mL). Across all age groups, the limits of agreement remained relatively wide, indicating substantial variability regardless of age (Table 2 and Figure 3).

## 4. Discussion

The study assessed agreement and reliability between a DIS named Breathive and the VIF (Voldyne device) across age groups from 7 to 80 years, with equal numbers of male and female participants. The findings demonstrated that the Breathive device exhibited good overall reliability (ICC = 0.868). However, Bland–Altman analysis revealed wide limits of agreement, suggesting considerable variability between the two devices and limited interchangeability in clinical practice.

The findings of the present study support the growing interest in incorporating digital technologies into incentive spirometry to overcome the limitations of conventional devices. Burns et al. reported that traditional incentive spirometers lack data recording, limiting objective assessment of patient adherence and clinical effectiveness [26]. To address this issue, the authors developed an electronic add-on device using reflective optical sensors to digitally capture inspiratory volume and flow from a conventional Voldyne incentive spirometer. Their study demonstrated that converting mechanical respiratory signals into digital data is feasible and may improve objective monitoring of respiratory rehabilitation. Similarly, the Breathive, which is a DIS, was developed to overcome the limitations of conventional incentive spirometers by providing real-time digital measurements of inspiratory volume and flow. However, unlike the study by Burns et al. [26], which primarily focused on device development and usability testing, the present study evaluated the agreement and reliability of the DIS across a wide age range (7–80 years). This is clinically important because respiratory physiology, lung capacity, and inspiratory performance vary considerably across different stages of life.

Age-related differences in lung volume are well established, with lung capacity increasing during growth and reaching a peak in early adulthood, followed by a gradual decline with aging [27]. These physiological changes may influence both inspiratory performance and measurement variability. Therefore, this might be affecting the agreement and reliability between devices across different age groups. Additionally, the highest reliability was observed in older adults (60–80 years), whereas adolescents (13–17 years) demonstrated the lowest ICC values. This variability may be attributed to differences in breathing patterns, effort consistency, and coordination across age groups. In younger participants, inconsistent inspiratory effort may have contributed to greater measurement variability [28,29]. Interestingly, the smallest bias was noted in the youngest group (7–12 years), whereas larger positive biases were found in adult groups, particularly those aged 36–59 years. This may reflect differences in lung capacity, inspiratory flow rates, and the mechanical characteristics of the devices. Higher inspiratory volumes are typically associated with increased airflow velocity, which may influence measurement accuracy, particularly in flow-based systems [29]. The increasing variability observed at higher inspiratory volumes suggests the presence of proportional bias, where measurement error increases with the magnitude of the measurement, as described in Bland–Altman analysis [24,30].

Several factors may explain the observed differences between devices. Differences in measurement principles, including flow-based sensing in the Breathive device versus volume displacement in the Voldyne, may contribute to systematic variation [31]. The Breathive is principally achieved by using the integration of a differential pressure-based flow sensor within the measurement system, which may introduce additional airflow resistance and pressure drop; therefore, this could alter inspiratory dynamics and slightly affect the volume measured by the volume-oriented device [30]. In addition, the resistive device, airflow dynamics, and patient technique may influence measurement outcomes, as respiratory measurements are highly effort-dependent and sensitive to breathing patterns [29,32]. The use of flow-based sensing may increase measurement noise due to airflow turbulence, pressure fluctuations, and electronic signal amplification. Therefore, inspiratory volume in the Breathive is derived from temporal integration of the airflow signal; such noise may accumulate and contribute to increased variability and wider limits of agreement. Further, the Voldyne device itself has limited measurement resolution, as it relies on a mechanical scale with relatively coarse gradations and requires visual estimation between markings. This may introduce observer-dependent variability and reduce measurement precision [12]. Altogether, these factors—including airflow resistance, signal noise, and limited resolution—may explain the observed bias and wide limits of agreement between the two devices.

The study has some limitations that might potentially affect the outcomes of the study. First, age was treated as a categorical variable, which may reduce statistical power and obscure variability within groups compared with treating age as a continuous variable. Consequently, future studies with larger sample sizes should consider regression-based analyses that treat age as a continuous variable to further investigate age-related influences on measurement agreement and reliability. A calibration against a laboratory-standard spirometer was not performed, which may limit the generalizability of the findings. Further, measurement discrepancies between the two devices may partly be attributed to differences in display resolution and reading precision. The integration of a flow-based sensor may have introduced additional resistance and measurement noise, and inspiratory volume in the digital device was estimated indirectly from airflow. In addition, the limited resolution of the Voldyne device may have contributed to measurement variability. Although the Voldyne provides volume markings, its scale is relatively coarse, requiring clinicians to estimate values between marked levels. This may introduce observer-dependent variability and reduce measurement accuracy. In contrast, the Breathive provides real-time numerical output, allowing for more precise and objective recording of inspiratory volume. The continuous digital display reduces the need for estimation and may improve measurement consistency. This difference in resolution and data presentation could contribute to the observed variability between the two devices. Therefore, the limited resolution of the Voldyne may introduce systematic and random measurement errors, whereas the Breathive device, with its real-time quantitative output, may enhance precision and reduce observer-related bias. Future studies should investigate the performance of the Breathive in clinical populations, such as patients with respiratory disease or postoperative conditions. Although the Breathive device demonstrated good reliability in all ages, the present study did not compare the Breathive device with a gold standard, such as pulmonary function testing. In addition, the order of device placement may influence inspiratory performance and measured inspiratory volume by altering airflow resistance, pressure drop, and airflow dynamics within the respiratory circuit. These factors could potentially affect the participant’s ability to generate inspiratory flow and achieve maximal inspiratory volume during testing. Finally, the Breathive estimates inspiratory volume indirectly from airflow measurements by integrating flow over time, whereas the Voldyne device measures volume directly through volume displacement. This difference in measurement principles may introduce additional sources of error, as small inaccuracies in flow measurement can accumulate during integration and affect volume estimation [33]. This approach suits research or digital spirometers with logging but demands validation against gold-standard volume measures [34].

## 5. Conclusions

In conclusion, to our knowledge, this is one of the first studies to evaluate the agreement and reliability of a DIS across a broad age range spanning from childhood to older adulthood. The Breathive demonstrated good reliability but limited agreement with the standard Voldyne device. Basically, incentive spirometers are primarily designed to encourage sustained deep inspiration, improve lung expansion, and enhance pulmonary rehabilitation rather than serve as highly precise pulmonary function testing. In addition, incentive spirometry is often based on the ability to promote repeated maximal inspiratory efforts and monitor relative changes over time rather than obtain exact pulmonary volume measurements. In addition, the limits of agreement between devices were relatively wide between the Breathive device and the Voldyne device. Therefore, the Breathive should not be considered interchangeable with standard pulmonary function testing for precise measurement, but it may serve as a useful tool for monitoring inspiratory performance and supporting pulmonary rehabilitation.

## Figures and Tables

**Table 1:** Characteristic data of the participants.

	Total (N = 150)	7–12 (*n* = 30)	13–17 (*n* = 30)	18–35 (*n* = 30)	36–59 (*n* = 30)	60–80 (*n* = 30)
Age (yr)	32.97 ± 22.01	9.00 ± 1.31	13.40 ± 0.67	28.27 ± 4.48	47.93 ± 6.76	65.6 ± 6.11
Breathive (mL/sec)	1292.55 ± 492.79	771.13 ± 359.67	1246.17 ± 302.48	1690.97 ± 403.67	1410.27 ± 351.87	1344.233 ± 524.69
Voldyne (mL/sec)	1243.86 ± 545.45	807.67 ± 304.46	1192.50 ± 404.08	1611.93 ± 576.72	1307.67 ± 515.22	1299.53 ± 572.22

**Table 2:** Agreement and reliability between the Breathive and Vodyle incentive spirometers across different age groups.

Age (Years)	N	ICC (2,1)	95%CI	Mean diff	LoA
7–80	150	0.868	0.821–0.903	48.69	566.63 to −469.24
7–12	30	0.882	0.769–0.942	−36.53	248.48 to −351.54
13–17	30	0.619	0.341–0.798	53.67	312.06 to 624.13
18–35	30	0.828	0.670–0.914	79.03	287.34 to 574.68
36–59	30	0.761	0.712–0.936	102.60	295.06 to 590.13
60–80	30	0.909	0.820–0.956	44.70	233.29 to 466.59

ICC, intraclass correlation coefficient; CI, confidence interval. LoA, limits of agreement. Note: ICCs were calculated using a two-way random-effects model with absolute agreement [ICC(2,1)]. ICC interpretation followed commonly accepted thresholds: poor (<0.50), moderate (0.50–0.75), good (0.75–0.90), and excellent (>0.90).

## Data Availability

The original contributions presented in this study are included in the article. Further inquiries can be directed to the corresponding author.

## Abbreviations

The following abbreviations are used in this manuscript:

ICC	intraclass correlation coefficients
DIS	digital incentive spirometer
VIS	volume-oriented incentive spirometer
FIS	flow-oriented incentive spirometer
LoA	limits of agreement
mL	milliliter
